# Livestock-associated Methicillin-Resistant *Staphylococcus aureus* Sequence Type 398 in Humans, Canada

**DOI:** 10.3201/eid1604.091435

**Published:** 2010-04

**Authors:** George R. Golding, Louis Bryden, Paul N. Levett, Ryan R. McDonald, Alice Wong, John Wylie, Morag R. Graham, Shaun Tyler, Gary Van Domselaar, Andrew E. Simor, Denise Gravel, Michael R. Mulvey

**Affiliations:** National Microbiology Laboratory, Winnipeg, Manitoba, Canada (G.R. Golding, L. Bryden, M.R. Graham, S. Tyler, G. Van Domselaar, M.R. Mulvey); Saskatchewan Disease Control Laboratory, Regina, Saskatchewan, Canada (P.N. Levett, R.R. McDonald); Royal University Hospital, Saskatoon, Saskatchewan, Canada (A. Wong); Cadham Provincial Laboratories, Winnipeg (J. Wylie); Sunnybrook Health Sciences Centre, Toronto, Ontario, Canada (A.E. Simor); Public Health Agency of Canada, Ottawa, Ontario, Canada (D. Gravel)

**Keywords:** Methicillin-resistant Staphylococcus aureus, ST398, livestock associated, SCCmec, CRISPR, zoonoses, Canada, bacteria, expedited, research

## Abstract

Rates of colonization with livestock-associated methicillin-resistant *Staphylococcus aureus* (MRSA) sequence type 398 have been high for pigs and pig farmers in Canada, but prevalence rates for the general human population are unknown. In this study, 5 LA-MRSA isolates, 4 of which were obtained from skin and soft tissue infections, were identified from 3,687 tested MRSA isolates from persons in Manitoba and Saskatchewan, Canada. Further molecular characterization determined that these isolates all contained staphylococcal cassette chromosome (SCC) *mec*V, were negative for Panton-Valentine leukocidin, and were closely related by macrorestriction analysis with the restriction enzyme *Cfr91*. The complete DNA sequence of the SCC*mec* region from the isolate showed a novel subtype of SCC*mec*V harboring clustered regularly interspaced short palindromic repeats and associated genes. Although prevalence of livestock-associated MRSA seems to be low for the general population in Canada, recent emergence of infections resulting from this strain is of public health concern.

## Abstract 2

Recent emergence of infections resulting from this strain is of public health concern.

High prevalence of colonization with livestock-associated (LA) methicillin-resistant *Staphylococcus aureus* (MRSA) sequence type (ST) 398 among pigs and pig farmers was first reported in the Netherlands (*1*) and has since been identified in Canada (*2*) and the United States (*3*). In Canada, this LA-MRSA strain was identified in pigs and pig farmers in southwestern Ontario, where prevalence of MRSA colonization was 24.9% (71/285) and 20% (5/25), respectively (*2*). In the United States, nasal samples from 20 production system workers and 299 swine from 2 farms in Illinois and Iowa showed that 45% (9/20) and 49% (147/299), respectively, were colonized with LA-MRSA (*3*). Despite such high prevalence of MRSA colonization on these tested farms, to our knowledge, no human or animal infections resulting from LA-MRSA strains have been reported in North America.

To determine whether LA-MRSA has recently emerged in the general population of Canada, we identified human infections and colonizations associated with the LA-MRSA strain in Canada and molecularly characterized the isolates. We also identified a novel staphylococcal cassette chromosome (SCC) *mec*V subtype harboring clustered regularly interspaced short palindromic repeats (CRISPR) and CRISPR-associated genes (*cas*).

## Materials and Methods

A convenience sample, totaling 2,358 MRSA isolates from human specimens, was submitted to the National Microbiology Laboratory (NML) for *spa* typing, as described (*4*,*5*). During January 2007–October 2008, the Saskatchewan Disease Control Laboratory submitted 2,008 specimens; during October 2007–August 2008, the Cadham Provincial Laboratory in Manitoba submitted 350 specimens. An additional 1,329 isolates from human specimens were *spa* typed by the Saskatchewan Disease Control Laboratory.

Given the client base of the Cadham Provincial Laboratory, most of these isolates would have originated from colonized and infected persons living in the community or in personal-care homes or from persons hospitalized in smaller community hospitals, whereas, for surveillance purposes, the Saskatchewan Disease Control Laboratory receives isolates from all colonized and infected persons across the province. Detailed information regarding why cultures were taken (e.g., screening admissions, outbreak investigations) and other clinical and epidemiologic data were limited. Isolates typed in this study represented ≈17% of all MRSA isolates from persons in Manitoba and ≈66% of all MRSA isolates from persons in Saskatchewan within the study period. An additional isolate was sent to the NML from Sunnybrook Health Sciences Centre in Ontario for reference purposes.

Isolates with *spa* types associated with ST398 were confirmed by multilocus sequence typing; tested for Panton-Valentine leukocidin toxin, *mec*A, and *nuc* genes; and typed for SCC*mec* as described (*6*–*9*). Pulsed-field gel electrophoresis (PFGE) of *Sma*I- or *Cfr*91-digested genomic DNA was conducted as described (*10*). Antimicrobial drug susceptibility testing was conducted by using standard broth microdilution panels according to Clinical and Laboratory Standards Institute guidelines (*11*). Breakpoints for fusidic acid and mupirocin resistance, which were not provided in the guidelines, were as described (*12*,*13*).

A fosmid library was constructed by cloning sheared genomic DNA from *S. aureus* isolate 08 BA 02176 into the pCC2FOS vector. The fosmid clones were screened by PCR to identify specific genes *orfX*, *mecA*, and the chromosomal region located downstream of SCC*mec*. Fosmid clone 1G1 was identified and contained the entire SCC*mec* region of the 08 BA 02176 isolate. Colonies were prepared by using the CopyControl Fosmid Library Production Kit (Epicenter Biotechnologies, Madison, WI, USA) according to the manufacturer’s instructions. Fosmid DNA was column purified by using a QIAGEN Plasmid Mini Kit (QIAGEN, Valencia, CA, USA).

DNA sequencing was performed on the ABI3730xl genetic analyzer (Applied Biosystems, Foster City, CA, USA). Staden (Pregap4) software was used to prepare trace data for sequence assembly (*14*). Sequencing reads were assembled by using the Staden Gap4 program. Gap closure was achieved by primer walking and long-range PCR. Specific primers were designed near the ends of neighboring contigs (contiguous sequence of DNA created by overlapping sequenced fragments of a chromosome), and PCRs were performed with chromosomal template DNA. Regions containing putative frameshifts and point mutations were resequenced to verify the fidelity of the sequence.

Annotation and data mining of the *S. aureus* 08 BA 02176 1G1 fosmid clone sequence were performed by using the GenDB version 2.2 annotation tool (*15*). Putative protein coding sequences were determined according to coding sequence predictions of Glimmer, which is integrated into the GenDB package. Similarity searches were performed by using BLASTN and BLAST2P (www.ncbi.nlm.nih.gov/blast/Blast.cgi) against the nonredundant nucleotide and protein databases, respectively. Additionally, a BLAST2P search was performed against the databases nr (ftp://ftp.ncbi.nlm.nih.gov/blast/db/), SWISS-PROT (www.expasy.ch/sprot/), and KEGG-Genes (ftp://ftp.genome.jp/pub/kegg/genes/); the protein family databases Pfam (http://pfam.sanger.ac.uk/) and TIGRFAM (www.jcvi.org/cms/research/projects/tigrfams/overview/); and predictive signal peptide (Signal P [www.cbs.dtu.dk/services/SignalP/]) and transmembrane helix analysis (TMHMM [www.cbs.dtu.dk/services/TMHMM/]), the nonredundant database on protein level. An automatic functional annotation was followed by a manual annotation of each predicted gene.

## Results

### LA-MRSA Characterization

A total of 3,687 MRSA isolates were examined; 5 contained ST398-associated *spa* types (4 t034 and 1 t1250). The additional isolate submitted to NML by Sunnybrook Health Sciences Centre in Ontario, isolate T40929, also contained a t034 *spa* type. Further molecular characterization of these 6 isolates determined that they were all ST398, SCC*mec*V, and negative for the Panton-Valentine leukocidin–encoding genes (Table 1). Of the 6 isolates, 5 were resistant to tetracycline, but all were susceptible to the other 12 antimicrobial drugs tested (Table 2).

**Table 1:** Characteristics of methicillin-resistant *Staphylococcus aureus* sequence type 398 novel staphylococcal cassette chromosome *mec*V subtype isolates, Canada*

Isolate	Collection date	Patient age, y/sex	Region and province	Specimen collection site	*spa* type
07 BA 06477	2007 Feb 27	26/F	Saskatoon, SK	Nasal screen	t034
08 BA 02176	2008 Jan 15	71/F	Sunrise, SK	Leg swab	t034
08 BA 08100	2008 Mar 4	51/M	Five Hills, SK	Left shin open abrasion	t1250
08 BA 13895	2008 Apr 25	79/M	Kelsey Trail, SK	Left hip swab	T034
08 BA 22334	2008 Jul 9	70/M	Prince Albert Parkland, SK	Right leg swab	T034
T40929	2007 Dec 11	59/M	Durham, ON	Nasal and tracheostomy screen	T034

*All isolates were Panton-Valentine leukocidin negative. SK, Saskatchewan; ON, Ontario.

**Table 2:** Antimicrobial drug susceptibility of the clinical isolates of methicillin-resistant *Staphylococcus aureus* sequence type 398, Canada, 2008*

Drug	Susceptibility, μg/mL					
	07 BA 06477	08 BA 02176	08 BA 08100	08 BA 13895	08 BA 22334	T40929
Clindamycin	<0.25	<0.25	<0.25	<0.25	<0.25	<0.25
Vancomycin	0.5	0.5	0.5	0.5	0.5	0.5
Erythromycin	0.5	0.5	0.5	0.5	0.5	0.5
SXT	<0.25	<0.25	<0.25	<0.25	<0.25	<0.25
Synercid	0.5	1	<0.25	<0.25	<0.25	<0.25
Nitrofurantoin	<32	<32	<32	<32	<32	<32
Tetracycline	>16	>16	>16	≤2	>16	>16
Ciprofloxacin	0.5	0.25	0.5	0.25	0.5	0.5
Rifampin	<0.25	<0.25	<0.25	<0.25	<0.25	<0.25
Fusidic acid	0.25	0.12	0.25	0.12	0.12	0.12
Linezolid	2	2	2	1	0.5	0.5
Gentamicin	1	1	1	1	≤0.5	1
Mupirocin	0.5	0.25	<0.12	<0.12	0.25	<0.12

*SXT, sulfamethoxazole/trimethoprim.

**Figure 1:**
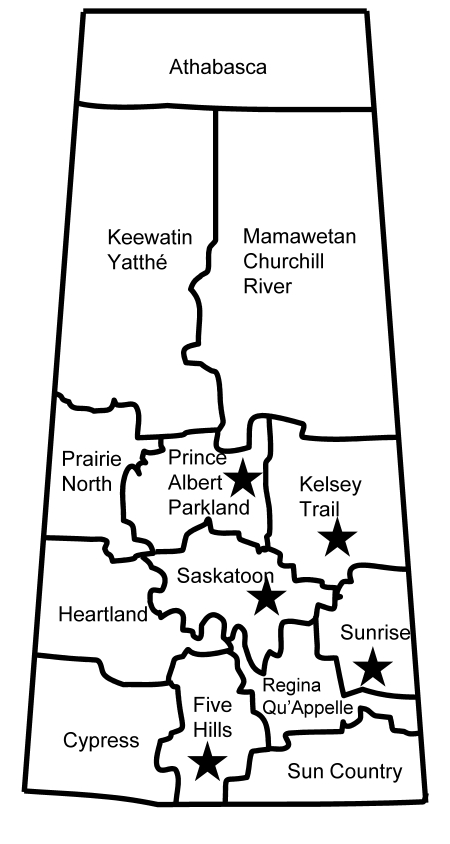
Geographic distribution of 5 livestock-associated methicillin-resistant *Staphylococcus aureus* isolates (stars) from humans, Saskatchewan, January 2007–October 2008.

From the surveillance in Manitoba and Saskatchewan, patient information was limited and showed no geographic links (all 5 persons resided in different health regions but were all within the southeastern portion of Saskatchewan) (Figure 1). Of the 5 isolates, 4 were obtained from infected persons (average age 67.8 years, range 51–79 years) (Table 1). The earliest identified LA-MRSA isolate (08 BA 2176) associated with an infection was obtained from a postoperative surgical site. Further follow-up was not possible because of the patient’s health problems. This patient is unlikely to have had any recent direct contact with livestock because she had been confined to her home with limited mobility for several years before her hospitalization. Additional nasal swabs from this patient remained positive for this strain for at least 7 months. Additional clinical and epidemiologic information for the remaining 3 patients with skin and soft tissue or wound infections were limited (Table 1).

The isolate submitted to the NML by Sunnybrook Health Sciences Centre, outside the surveillance program, was from a 59-year-old man from Ontario. He had been hospitalized in December 2007 for treatment of metastatic squamous cell carcinoma of the larynx. In the previous year, he had undergone a total laryngectomy, neck node dissection, and tracheostomy. A MRSA isolate was recovered from screening specimens from his nose and the tracheostomy site that had no indication of infection. He was unaware of any animal contact and had no history of exposure to pigs or pig farms. A review of the medical records and standard epidemiologic investigations determined that this was not a nosocomial or healthcare-associated isolate.

**Figure 2:**
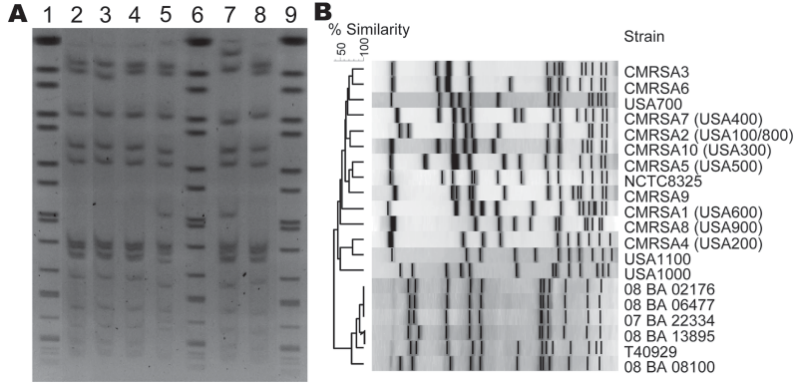
A) Pulsed-field gel electrophoresis (PFGE) of *Cfr*91-digested livestock-associated methicillin-resistant *Staphylococcus aureus* (MRSA). Lanes 1, 6, and 9, universal standard *Salmonella* Braenderup H9812; Lane 2, 08 BA 02176; Lane 3, 08 BA 13895; Lane 4, 07 BA 06477; Lane 5, T40929; Lane 7, 08 BA 08100; Lane 8, 07 BA 22334. B) PFGE dendrogram comparing the *Cfr*91 fingerprint patterns of 6 livestock-associated MRSA isolates from humans in Canada with the *Sma*I fingerprints of other human epidemic strains of MRSA circulating in Canada.

The 6 LA-MRSA isolates were nontypeable by PFGE using *Sma*I. However, PFGE using the neoschizomer *Cfr*91 showed that the 6 LA-MRSA isolates were closely related (Figure 2, panel A). Control MRSA strains digested individually with either *Cfr*91 or *Sma*I showed no differences in fingerprint banding patterns when the 2 enzymes were compared (data not shown), which enabled comparisons of the PFGE patterns obtained for the LA-MRSA isolates with those of other epidemic MRSA strains from hospitals and communities in Canada. No close relatedness was found (>7 bands difference; <80% similarity) between the LA-MRSA isolates and any other epidemic MRSA strain circulating in Canada (Figure 2, panel B).

### SCC*mec* Characterization

**Figure 3:**
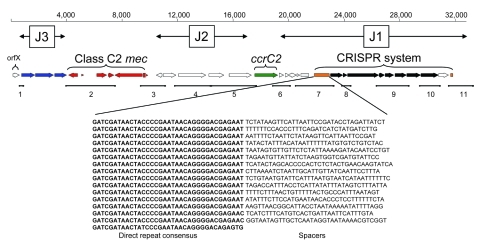
Schematic of the novel staphylococcal cassette chromosome (SCC) *mec*V subtype and DNA sequence of the clustered regularly interspaced short palindromic repeat (CRISPR) array identified in *Staphylococcus aureus* isolate 08 BA 02176. Red and green arrows represent *mec* and *ccr* complexes, respectively. Blue arrows represent 3 open reading frames (ORFs) in the J3 region sharing sequence identity with chromosomal genes of *S. epidermidis* RP62A. Orange boxes indicate confirmed and questionable CRISPRs. Black arrows represent CRISPR-associated genes. Location of primer sets used for coverage of this SCC*mec* element are numbered 1–11 (Table 4) and illustrated as solid lines. Shown below the schematic is the DNA sequence of the confirmed 1,107-bp CRISPR array in the J1 region, which provides the 36-bp direct repeat consensus (**boldface**) and the variable 15 spacer sequences.

DNA sequencing of the entire SCC*mec* element from isolate 08 BA 02176 showed a 32,369-bp element integrated at the 3′ end of *orf*X containing 30 putative open reading frames (ORFs) (Figure 3; Table 3). This element carried a class C2 *mec* complex, which putatively contained a nonfunctional IS431 transposase and a type 5 *ccr* gene complex (*ccr*C2). Other than *mecA*, no additional antimicrobial drug resistance genes were identified within this element.

**Table 3:** Open reading frames of the novel staphylococcal cassette chromosome *mec*V subtype in methicillin-resistant *Staphylococcus aureus* isolate 08 BA 02176, from woman in Canada, 2008*

ORF	Location, bp†	Predicted gene size, bp	Gene‡	Product description	Amino acid identity, %§	GenBank accession no.
Sk01	1–480	480	*orfX*	Conserved hypothetical protein	100	gb|ACC96139.1|
Sk02	609–1595	987	None	ADP-ribosylglycohydrolase	99	gb|AAW53059.1|
Sk03	1614–2948	1335	None	Permease for cytosine/purines; uracil; thiamine; allantoin	98	gb|AAW53058.1|
Sk04	2999–3883	885	None	Ribokinase	98	gb|AAW53057.1|
Sk05	(4013–4687)	675	*tnp*	Transposase for IS431	100	dbj|BAD24823.1|
Sk06	4945–5112	168	None	HMG-CoA synthase truncation	100	ref|YP_184940.1|
Sk07	6029–6772	744	*ugpQ*	Glycerophosphoryl diester phosphodiesterase	100	ref|NP_370563.1|
Sk08	6869–7297	429	*maoC*	Hypothetical protein	100	ref|YP_184943.1|
Sk09	(7343–9349)	2007	*mecA*	Penicillin-binding protein 2′	100	dbj|BAG06200.1|
Sk10	9449–9559	Unknown	ψ*mecR1*	Truncated signal transducer protein MecR1	100	ref|YP_252007.1|
Sk11	9597–9740	144	ψ*tnp*	Partial transposase for insertion sequence–like element IS431mec	100	dbj|BAH57698.1|
Sk12	(10331–10759)	429	None	Hypothetical protein	100	dbj|BAD24829.1|
Sk13	10840–11769	930	None	Hypothetical protein	100	gb|ACL99839.1|
Sk14	11931–13919	1989	None	Hypothetical protein	100	gb|ACL99840.1|
Sk15	14114–15223	1110	None	Hypothetical protein	100	gb|ACL99841.1|
Sk16	15584–17200	1617	None	Hypothetical protein	100	gb|ACL99843.1|
Sk17	17425–19104	1680	*ccrC*	Cassette chromosome recombinase C	100	gb|ACL99844.1|
Sk18	19193–19531	339	None	Hypothetical protein	100	gb|ACL99845.1|
Sk19	19625–19936	312	None	Hypothetical protein	100	gb|ACL99846.1|
Sk20	19951–20454	504	None	Hypothetical protein	100	gb|ACL99847.1|
Sk21	20469–20690	222	None	Hypothetical protein	100	gb|ACL99848.1|
Sk22	(20853–21256)	403	ψ*hsdR*	Truncated *hsdR*	92	dbj|BAG71456.1|
Sk23	22888–23793	906	*cas1*	CRISPR–associated Cas1 family protein	91	gb|AAW53332.1|
Sk24	23793–24098	306	*cas2*	CRISPR-associated protein Cas2	87	gb|AAW53331.1|
Sk25	24112–26385	2274	*csm1*	CRISPR-associated protein; Csm1 family	92	gb|AAW53330.1|
Sk26	26388–26813	426	*csm2*	CRISPR-system related protein	94	gb|AAW53329.1|
Sk27	26815–27459	645	*csm3*	CRISPR-associated RAMP protein	96	gb|AAW53328.1|
Sk28	27530–28378	849	*csm4*	CRISPR-associated RAMP protein	91	gb|AAW53327.1|
Sk29	28381–29403	1023	*csm5*	CRISPR-associated Csm5 family protein	92	gb|AAW53326.1|
Sk30	29403–30671	1269	*csm6*	CRISPR-associated protein (Cas_Csm6)	73	gb|AAW53325.1|
Sk31	30668–31402	735	*cas6*	CRISPR-associated protein C	86	gb|AAW53324.1|

*ORF, open reading frame; CRISPR, clustered regularly interspaced short palindromic repeats. †Parentheses indicate complement sequences. ‡None indicates no name given. §Comparisons of translated query versus protein databases was determined by using BLASTX 2.2.21 (www.ncbi.nlm.nih.gov/blast/Blast.cgi).

The first unique feature of this SCC*mec*V element included 3 ORFs in the J3 region sharing high sequence identity with ORFs from *S. epidermidis* RP62A (GenBank accession no. CP000029), which included an ADP-ribosylglycohydrolase, a permease for cytosine/purines, and a ribokinase (Table 3). A second unique feature was a CRISPR array, identified by using CRISPRFinder (*16*), in the J1 region, which appears to have replaced the type 1 restriction modification system (*hsdR*, *hsdS*, *hsdM*) through recombination. The CRISPR array (1,107 bp) contained a 36-bp direct repeat consensus and 15 spacers of variable sequence and length (33–38 bp) (Figure 3). Downstream of this CRISPR array was a combination of putative CRISPR-associated (*cas*) genes, sharing sequence identity with those previously identified in *S. epidermidis* RP62A. This array was followed by a second questionable CRISPR array (183 bp) containing a 38-bp direct repeat consensus and 2 spacers of variable sequence (Figure 3; Table 3).

Design of primers spanning the entire SCC*mec* element was based on the DNA sequence obtained from 08 BA 02176 (Figure 3; Table 4). PCR of these select regions produced amplicons of expected size for 3 additional LA-MRSA isolates (07 BA 06477, 08 BA 13895, 08 BA 22334) but were negative for some of the J1 and J3 regions in 08 BA 08100 and T40929 (Table 4).

**Table 4:** Primers used for coverage of the novel SCC*mec*V subtype in methicillin-resistant *Staphylococcus aureus* isolates, Canada, 2007–2008*

Primer set	Primer name	Primer 5′ → 3′	Expected amplicon size, bp	Reference position	SCC*mec*V found in isolate					
					08 BA 02176	T 49209	07 BA 06477	08 BA 13895	08 BA 08100	08 BA 22334
1	OrfX Adpr1	CATTTAAGATTATGCGTGGAG CATCTGTAAACTGTCCTTTGG	347	443–789	+	–	+	+	–	+
2	RibB2 MecaA1	TTGTATATGGGGAAACGAAG TGCCAAAATCTCAGGTAAAG	3623	3793–7415	+	–	+	+	–	+
3	MecB1 HypA1	CTTCACCATTATCGCTTTTAG ACCATTTTTCCCTGGATTAC	1842	9172–11013	+	+	+	+	+	+
4	Hyp3A1 Hyp1B1	CTTCCACGTATTGGTCTAGC AAGTGAACGCGAAAGATATAG	2671	11636–14306	+	+	+	+	+	+
5	Hyp3B1 CcrCA2	GCTAGACCAATACGTGGAAG TTTTACCTGAAATGCCTGAG	3301	14287–17587	+	+	+	+	+	+
6	CcrCB1 Hyp6A1	ATGAAATGGATAGCGAAATG TTGAGTAAGTAGCGGTGTTG	1330	18695–20024	+	+	+	+	+	+
7	Hyp6B1 Crspr1A1	TGAGCAAGTGATGGAAATG CTTTGAATCCTTTGAAGACG	2835	20331–23165	+	–	+	+	–	+
8	Crspr1B1 Crspr3A1	AAAAAGTGGTGAGGTTACTTG CTCGTCTATCAATACCACTCG	711	23675–24385	+	–	+	+	–	+
9	Crspr3B1 Crspr7A1	AACAGATGAACACGGAAAAG TTGGTGGGTATCTCAAAAAG	2417	26166–28582	+	–	+	+	–	+
10	Crspr7B1 Hyp11A1	GCCTTCTAACGTACCAGTTG TTGCTTCAATGGACTATAAGC	1511	29289–30820	+	–	+	+	–	+
11	Hyp11B1 Hyp12A1	TTAGGCATGGGGAAATATAG GTCGCAATGTTTTGAAGTG	1622	31373–	+	–	+	+	–	+

*SCC*mec,* staphylococcal cassette chromosome *mec*V subtype; +, positive; –, negative. Testing by PCR.

## Discussion

The high prevalence of LA-MRSA colonization of pigs and pig farmers in Canada (*2*) and the United States (*3*) and this report of human infections suggest that this LA-MRSA strain from Canada poses potential public and occupational health concern in North America. This strain has been associated with various types of infections in pigs (*17*,*18*) and humans (*19*,*20*) and is transmissible from animal patients to veterinary workers (*21*), healthcare workers (*22*), and family members (*1*). Evidence also suggests that this strain might be spreading from animals to the environment, which may facilitate the colonization or infection of persons who are not involved in animal husbandry (*23*). Whereas in 2006 in the Netherlands LA-MRSA accounted for >20% of all MRSA isolated (*24*), carriage of this strain in the general population of 2 provinces in Canada (Manitoba and Saskatchewan) appears rare (0.14%). This difference could be attributed to the substantially higher density of pigs in the Netherlands (1,244 pigs/km^2^) than in Manitoba (55 pigs/km^2^), Saskatchewan (6 pigs/km^2^), and Ontario (91 pigs/km^2^) (www.agriculture.gov.sk.ca/Pig_Densities). It is also plausible that the much lower proportions of LA-MRSA in Canada, relative to a country with low MRSA endemicity such as the Netherlands, is attributable to competition with other highly successful human epidemic MRSA clones circulating in Canada, including CMRSA2 (USA200/800), CMRSA7 (USA400), and CMRSA10 (USA300) (*25*,*26*).

The tested LA-MRSA isolates were highly susceptible to most classes of antimicrobial drugs, except β-lactams and tetracyclines, the latter of which has been attributed to its high usage in animal husbandry (*27*). The complete sequence of the SCC*mec* region showed a novel SCC*mec*V subtype sharing sequence identity in its J1 and J3 regions with chromosomal genes in the *S. epidermidis* RP62A chromosome (GenBank accession no. CP000029), including a CRISPR system. CRISPRs and associated *cas* genes are present in many other bacterial (≈40%) and archaeal (≈90%) genomes (*28*,*29*) and have been shown to be involved in sequence-directed immunity against phages (*30*,*31*) and plasmids (*32*). The resistance to plasmids and phages encoded by this system could explain why many of these ST398-MRSA-V strains contain fewer antimicrobial drug resistance genes and phage-encoded virulence factors than do other epidemic MRSA strains (*33*,*34*). The origin of this CRISPR system is unknown, but the propagation of CRISPR loci throughout prokaryote genomes has been proposed to occur through horizontal gene transfer by conjugation of megaplasmids >40 kb (*35*). Because the CRISPR system identified in this study is encoded within a putative mobile genetic element, we propose that an additional mechanism of mobilization to other methicillin-susceptible *Staphylococcus* spp. is plausible.

This novel subtype of SCC*mec*V was found in only 4 of the 6 LA-MRSA isolates identified in this study. One isolate not containing this novel SCC*mec* subtype (08 BA 08100) could also be distinguished by a different but closely related *spa* type (t1250) (Table 1) and variant PFGE fingerprint (Figure 2) when compared with the other LA-MRSA isolates, which suggests that at least 2 epidemiologically different strains of LA-MRSA circulate in Saskatchewan. The other LA-MRSA isolate that did not contain this novel SCC*mec* element was obtained in Ontario. However, this isolate was the same *spa* type (t034) and was closely related, according to PFGE, to the LA-MRSA isolates identified in Saskatchewan. Therefore, in addition to PFGE and *spa* typing, SCC*mec* subtyping could provide a useful epidemiologic tool for surveillance, outbreak investigations, or traceability studies of this emerging strain. For detection of this SCC*mec*V subtype (tentatively designated V.2.1.2; Vb), we propose using primer set 1 (spanning orfX into Sk02 in the J3 region) and primer set 7 (spanning Sk20 into cas1 in the J1 region) (Table 4).

Visual comparison of PFGE fingerprints from this study with those reported from patients from the Dominican Republic and the United States (northern Manhattan, New York, NY) (*36*), showed substantial variations in fingerprint patterns, as well as related but different *spa* types. These variations suggest further molecular and geographic diversity of these LA-MRSA strains on a global scale.

Because cases of LA-MRSA infections have only recently been identified in Canada, additional surveillance efforts are required to monitor the emergence and clinical relevance of this MRSA strain in Canada, including communities, the environment, livestock, farmers, and production facility workers. Whether these strains pose a major threat to human health in light of the low livestock density and continued spread of epidemic hospital and community strains of MRSA in Canada remains unknown.

## References

[1] Voss A, Loeffen F, Bakker J, Klaassen C, Wulf M. Methicillin-resistant *Staphylococcus aureus* in pig farming. Emerg Infect Dis. 2005;11:1965–6. 16485492 10.3201/eid1112.050428 PMC3367632

[2] Khanna T, Friendship R, Dewey C, Weese JS. Methicillin resistant *Staphylococcus aureus* colonization in pigs and pig farmers. Vet Microbiol. 2008;128:298–303. 10.1016/j.vetmic.2007.10.006 18023542

[3] Smith TC, Male MJ, Harper AL, Kroeger JS, Tinkler GP, Moritz ED, Methicillin-resistant *Staphylococcus aureus* (MRSA) strain ST398 is present in midwestern U.S. swine and swine workers. PLoS One. 2009;4:e4258. 10.1371/journal.pone.0004258 19145257 PMC2626282

[4] Golding GR, Campbell JL, Spreitzer DJ, Veyhl J, Surynicz K, Simor A, ; Canadian Nosocomial Infection Surveillance Program. A preliminary guideline for the assignment of methicillin-resistant *Staphylococcus aureus* to a Canadian pulsed-field gel electrophoresis epidemic type using *spa* typing. Can J Infect Dis Med Microbiol. 2008;19:273–81. 19436507 10.1155/2008/754249 PMC2604773

[5] Harmsen D, Claus H, Witte W, Rothgänger J, Claus H, Turnwald D, Typing of methicillin-resistant *Staphylococcus aureus* in a university hospital setting using a novel software for *spa*-repeat determination and database management. J Clin Microbiol. 2003;41:5442–8. 10.1128/JCM.41.12.5442-5448.2003 14662923 PMC309029

[6] Enright MC, Day NP, Davies CE, Peacock SJ, Spratt BG. Multilocus sequence typing for characterization of methicillin-resistant and methicillin-susceptible clones of *Staphylococcus aureus.* J Clin Microbiol. 2000;38:1008–15. 10698988 10.1128/jcm.38.3.1008-1015.2000 PMC86325

[7] McDonald RR, Antonishyn NA, Hansen T, Snook LA, Nagle E, Mulvey MR, Development of a triplex real-time PCR assay for detection of Panton-Valentine leukocidin toxin genes in clinical isolates of methicillin-resistant *Staphylococcus aureus.* J Clin Microbiol. 2005;43:6147–9. 10.1128/JCM.43.12.6147-6149.2005 16333116 PMC1317231

[8] Zhang K, McClure JA, Elsayed S, Louie T, Conly JM. Novel multiplex PCR assay for characterization and concomitant subtyping of staphylococcal cassette chromosome *mec* types I to V in methicillin-resistant *Staphylococcus aureus.* J Clin Microbiol. 2005;43:5026–33. 10.1128/JCM.43.10.5026-5033.2005 16207957 PMC1248471

[9] Kondo Y, Ito T, Ma XX, Watanabe S, Kreiswirth BN, Etienne J, Combination of multiplex PCRs for SCC*mec* type assignment: rapid identification system for *mec, ccr*, and major differences in junkyard regions. Antimicrob Agents Chemother. 2007;51:264–74. 10.1128/AAC.00165-06 17043114 PMC1797693

[10] Mulvey MR, Chui L, Ismail J, Louie L, Murphy C, Chang N, ; Canadian Committee for the Standardization of Molecular Methods. Development of a Canadian standardized protocol for subtyping methicillin-resistant *Staphylococcus aureus* using pulsed-field gel electrophoresis. J Clin Microbiol. 2001;39:3481–5. 10.1128/JCM.39.10.3481-3485.2001 11574559 PMC88375

[11] Clinical and Laboratory Standards Institute. Performance standards for antimicrobial susceptibility testing; 17th informational supplement. Document M100–S17. Wayne (PA): The Institute; 2007.

[12] Skov R, Frimodt-Moller N, Espersen F. Correlation of MIC methods and tentative interpretive criteria for disk diffusion susceptibility testing using NCCLS methodology for fusidic acid. Diagn Microbiol Infect Dis. 2001;40:111–6. 10.1016/S0732-8893(01)00262-0 11502378

[13] Walker ES, Levy F, Shorman M, David G, Abdalla J, Sarubbi FA. A decline in mupirocin resistance in methicillin-resistant *Staphylococcus aureus* accompanied administrative control of prescriptions. J Clin Microbiol. 2004;42:2792–5. 10.1128/JCM.42.6.2792-2795.2004 PMC427807 15184473

[14] Staden R, Beal KF, Bonfield JK. The Staden package. In: Misener S, Krawetz SA, editors. Computer methods in molecular biology, bioinformatics methods and protocols. Vol. 132. Totowa (NJ): The Humana Press Inc.; 1998. p. 115–130.

[15] Meyer F, Goesmann A, McHardy AC, Bartels D, Bekel T, Clausen J, GenDB—an open source genome annotation system for prokaryote genomes. Nucleic Acids Res. 2003;31:2187–95. 10.1093/nar/gkg312 12682369 PMC153740

[16] Grissa I, Vergnaud G, Pourcel C. CRISPRFinder: a web tool to identify clustered regularly interspaced short palindromic repeats. Nucleic Acids Res. 2007 Jul;35(Web Server issue):W52–7. 10.1093/nar/gkm360 PMC1933234 17537822

[17] Schwarz S, Kalec K, Strommenger B. Methicillin-resistant *Staphylococcus aureus* and *Staphylococcus pseudintermedius* detected in the Bft-GermVet monitoring programme 2004–2006 in Germany. J Antimicrob Chemother. 2008;61:282–5. 10.1093/jac/dkm487 18096559

[18] van Duijkeren E, Jansen MD, Flemming SC, de Neeling H, Wagenaar JA, Schoormans AH, Methicillin-resistant *Staphylococcus aureus* in pigs with exudative epidermitis. Emerg Infect Dis. 2007;13:1408–10. 18252124 10.3201/eid1309.061268 PMC2857271

[19] Witte W, Strommenger B, Stanek C, Cuny C. Methicillin-resistant *Staphylococcus aureus* ST398 in humans and animals, Central Europe. Emerg Infect Dis. 2007;13:255–8. 10.3201/eid1302.060924 17479888 PMC2725865

[20] Krziwanek K, Metz-Gercek S, Mittermayer H. Methicillin-resistant *Staphylococcus aureus* ST398 from human patients, upper Austria. Emerg Infect Dis. 2009;15:766–9. 10.3201/eid1505.080326 19402964 PMC2687006

[21] Wulf MW, Sørum M, van Nes A, Skov R, Melchers WJ, Klaassen CH, Prevalence of methicillin-resistant *Staphylococcus aureus* among veterinarians: an international study. Clin Microbiol Infect. 2008;14:29–34. 10.1111/j.1469-0691.2007.01873.x 17986212

[22] van Rijen MM, Van Keulen PH, Kluytmans JA. Increase in a Dutch hospital of methicillin-resistant *Staphylococcus aureus* related to animal farming. Clin Infect Dis. 2008;46:261–3. 10.1086/524672 18171259

[23] Gibbs SG, Green CF, Tarwater PM, Mota LC, Mena KD, Scarpino PV. Isolation of antibiotic-resistant bacteria from the air plume downwind of a swine confined or concentrated animal feeding operation. Environ Health Perspect. 2006;114:1032–7. 10.1289/ehp.8910 16835055 PMC1513331

[24] van Loo I, Huijsdens X, Tiemersma E, de Neeling A, van de Sande-Bruinsma N, Beaujean D, Emergence of methicillin-resistant *Staphylococcus aureus* of animal origin in humans. Emerg Infect Dis. 2007;13:1834–9. 18258032 10.3201/eid1312.070384 PMC2876750

[25] Simor AE, Ofner-Agostini M, Bryce E, McGeer A, Paton S, Mulvey MR; Canadian Hospital Epidemiology Committee and Canadian Nosocomial Infection Surveillance Program. Laboratory characterization of methicillin-resistant *Staphylococcus aureus* in Canadian hospitals: results of 5 years of national surveillance, 1995–1999. J Infect Dis. 2002;186:652–60. 10.1086/342292 12195352

[26] Christianson S, Golding GR, Campbell J. Canadian Nosocomial Infection Surveillance Program, Mulvey MR. Comparative genomics of Canadian epidemic lineages of methicillin-resistant *Staphylococcus aureus.* J Clin Microbiol. 2007;45:1904–11. 10.1128/JCM.02500-06 17428941 PMC1933033

[27] de Neeling AJ, van den Broek MJ, Spalburg EC, van Santen-Verheuvel MG, Dam-Deisz WDC, Boshuizen HC, High prevalence of methicillin resistant *Staphylococcus aureus* in pigs. Vet Microbiol. 2007;122:366–72. 10.1016/j.vetmic.2007.01.027 17367960

[28] Kunin V, Sorek R, Hugenholtz P. Evolutionary conservation of sequence and secondary structures in CRISPR repeats. Genome Biol. 2007;8:R61 . 10.1186/gb-2007-8-4-r61 17442114 PMC1896005

[29] Grissa I, Vergnaud G, Pourcel C. The CRISPRdb database and tools to display CRISPRs and to generate dictionaries of spacers and repeats. BMC Bioinformatics. 2007;8:172. 10.1186/1471-2105-8-172 17521438 PMC1892036

[30] Brouns SJ, Jore MM, Lundgren M, Westra ER, Slijkhuis RJ, Snijders AP, Small CRISPR RNAs guide antiviral defense in prokaryotes. Science. 2008;321:960–4. 10.1126/science.1159689 18703739 PMC5898235

[31] Barrangou R, Fremaux C, Deveau H, Richards M, Boyaval P, Moineau S, CRISPR provides acquired resistance against viruses in prokaryotes. Science. 2007;315:1709–12. 10.1126/science.1138140 17379808

[32] Marraffini LA, Sontheimer EJ. CRISPR interference limits horizontal gene transfer in staphylococci by targeting DNA. Science. 2008;322:1843–5. 10.1126/science.1165771 19095942 PMC2695655

[33] Monecke S, Jatzwauk L, Weber S, Slickers P, Ehricht R. DNA microarray-based genotyping of methicillin-resistant *Staphylococcus aureus* strains from eastern Saxony. Clin Microbiol Infect. 2008;14:534–45. 10.1111/j.1469-0691.2008.01986.x 18373691

[34] Walther B, Monecke S, Ruscher C, Friedrich AW, Ehricht R, Slickers P, Comparative molecular analysis substantiates zoonotic potential of equine methicillin-resistant *Staphylococcus aureus.* J Clin Microbiol. 2009;47:704–10. 10.1128/JCM.01626-08 19109463 PMC2650932

[35] Godde JS, Bickerton A. The repetitive DNA elements called CRISPRs and their associated genes: evidence of horizontal transfer among prokaryotes. J Mol Evol. 2006;62:718–29. 10.1007/s00239-005-0223-z 16612537

[36] Bhat M, Dumortier C, Taylor BS, Miller M, Vasquez G, Yunen J, *Staphylococcus aureus* ST398, New York City and Dominican Republic. Emerg Infect Dis. 2009;15:285–7. 10.3201/eid1502.080609 19193274 PMC2657615

